# An Insulin-like Growth Factor-1 Conjugated *Bombyx mori* Silk Fibroin Film for Diabetic Wound Healing: Fabrication, Physicochemical Property Characterization, and Dosage Optimization In Vitro and In Vivo

**DOI:** 10.3390/pharmaceutics13091459

**Published:** 2021-09-13

**Authors:** Meng-Jin Lin, Mei-Chun Lu, Yun-Chen Chan, Yu-Fen Huang, Hwan-You Chang

**Affiliations:** 1 Miaoli District Agricultural Research and Extension Station, Council of Agriculture, Executive Yuan, Miaoli 363201, Taiwan; Lmj@mdais.gov.tw (M.-J.L.); Lumj@mdais.gov.tw (M.-C.L.); Cyc@mdais.gov.tw (Y.-C.C.); 2 Institute of Molecular Medicine, National Tsing Hua University, Hsinchu 300044, Taiwan; 3 Department of Raw Material and Fiber, Taiwan Textile Research Institute, New Taipei City 236039, Taiwan; yfHuang.1413@ttri.com.tw

**Keywords:** silkworm, fibroin, insulin-like growth factor-1, diabetic wound, dosage-dependent effect

## Abstract

This study aimed to develop a silk fibroin (SF)-film for the treatment of chronic diabetic wounds. Silk fibroin was purified through a newly developed heating degumming (HD) process and casted on a hydrophobic surface to form SF-films. The process allowed the fabricated film to achieve a 42% increase in transparency and a 32% higher proliferation rate for BALB/3T3 fibroblasts compared to that obtained by conventional alkaline degumming treatment. Fourier transform infrared analysis demonstrated that secondary structure was retained in both HD- and alkaline degumming-derived SF preparations, although the crystallinity of beta-sheet in SF-film after the HD processing was slightly increased. This study also investigated whether conjugating insulin-like growth factor-1 (IGF-1) would promote diabetic wound healing and what the optimal dosage is. Using BALB/3T3 cells grown in hyperglycemic medium as a model, it was demonstrated that the optimal IGF-1 dosage to promote the cell growth was approximately 0.65 pmol. Further analysis of wound healing in a diabetic mouse model indicated that SF-film loaded with 3.25 pmol of IGF-1 showed significantly superior wound closure, a 13% increase at the 13th day after treatment relative to treatment with 65 pmol of free IGF-1. Improvement in diabetic wound healing was exerted synergistically by SF-film and IGF-1, as reflected by parameters including levels of re-epithelialization, epithelial tissue area, and angiogenesis. Finally, IGF-1 increased the epithelial tissue area and micro-vessel formation in a dose-dependent manner in a low dosage range (3.25 pmol) when loaded to SF-films. Together, these results strongly suggest that SF-film produced using HD and loaded with a low dosage of IGF-1 is a promising dressing for diabetic wound therapy.

## 1. Introduction

Diabetes mellitus is one of the most prevalent non-communicable diseases, affecting 422 million adults in 2014 and causing about 1.5 million deaths in 2012 [1]. Approximately 15% of diabetic patients suffer from chronic wounds or foot ulcers that frequently lead to limb amputation or even death [2,3]. In 2010, approximately 73,000 amputations were performed on diabetic patients with non-healing wounds in the United States [4]. Currently, the standard practices in diabetic wound management include surgical debridement, systematical glycemic control, wound off-loading, and dressings [5,6]. Although significant efforts have been exerted to improve diabetic wound healing, no satisfactory therapy has been developed thus far [7]. Therefore, more research on novel interventions to improve healing of diabetes-associated chronic wounds is urgently required.

Compared to wounds caused by trauma or burn, chronic diabetic wounds typically show a higher degree of aberrant angiogenesis and a lack of essential growth factors, such as insulin-like growth factors (IGFs) [8,9]. An early report has demonstrated that the distribution of IGF-1 is restricted to the stratum granulosum and spinosum of uninjured skin and is absent at the edge of diabetic foot ulcers, implicating its importance in healing chronic diabetic wounds [10]. Insulin-like growth factor-1 is a 7.6 kDa single-chain polypeptide with three intramolecular disulfide bridges. The IGF-1 receptor is a transmembrane receptor tyrosine kinase that exhibits auto-phosphorylation activity when activated by IGF-1 [11]. Activation of the IGF-1 receptor results in phosphorylation of insulin receptor substrates and Shc, thus initiating the cell proliferation pathway [12]. Furthermore, IGF-1 stimulates the Akt signaling pathway and promotes corneal epithelial cell growth for eye wound healing [13,14]. Although IGF-1 is a promising candidate for wound treatment , its application is significantly limited by the short half-life (<15 min) [15,16]. Therefore, development of a delivery system to maintain the activity of IGF-1 for a longer time is necessary for clinical application of the growth factor.

Silk fibroin (SF), from the common silkworm *Bombyx mori* L., is a biomaterial with excellent mechanical strength, biocompatibility, and biodegradability [17,18,19]. Silk fibroin is secreted from the posterior silk gland as a 2.3 MDa elementary unit, consisting of six sets of a disulfide-linked 350 kDa heavy-chain/24–26 kDa light-chain heterodimer and one molecule of fibrohexamerin [20]. *Bombyx mori* fibroin is mainly composed of glycine (43%), alanine (30%), and serine (12%) [21]. Silk fibroin has been demonstrated to be a promising material for sustained drug delivery through modifications [22]. Previous studies also indicated that SF not only has strong stimulatory effects on cell attachment and proliferation [23,24,25], but also inhibits wound scar formation in excisional or burn wounds [26,27]. The protein has been tested in several modalities, including film [28,29,30], sponge [31], hydrogel [32], and electrospun mat [33,34,35].

In our earlier study, we engineered a novel SF-film for drug delivery and verified its release profile [36]. Nevertheless, little is known about the effect of different manufacturing procedures on SF-films. In addition, although SF seems to be an excellent carrier of IGF-1 for wound healing, the dosage effect of combining silk fibroin and IGF-1 for clinical application has never been explored. Therefore, the aim of this study was to establish a simple-to-perform procedure to manufacture an advanced wound-dressing and characterize its physicochemical properties. The optimal dosage of IGF-1 complexed with the SF-film in wound repair was also evaluated. Finally, through in vivo testing, the most effective dosage of IGF-1 loaded in the SF-film for diabetic wound therapy is recommended.

## 2. Materials and Methods

### 2.1. Preparation of SF-Films

Silkworms were reared with mulberry leaves at 28 °C in our specifically designated facility (Taiwan Silkworm Germplasm, Miaoli District Agricultural Research and Extension Station, Taiwan). The harvested cocoons were degummed using either a previously described alkaline degumming (AD) procedure [17,19] by boiling in 0.02 M sodium carbonate solution, or a heating degumming (HD) procedure as described previously [36]. Briefly, cocoons were heated in deionized water (1% *w*/*v*) at 1.2 kg/cm^2^ pressure for 1, 2, and 3 h (designated HD1, HD2, and HD3) to remove sericin from raw silk. The SF fibers were dried at 50 °C for 24 h. The weight loss of the silk after degumming was determined using the formula, W_L_ = [(W_0_ − W_1_)/W_0_] × 100%, where W_L_ is the percentage of weight loss, W_0_ is the initial weight of the cocoon, and W_1_ is the final weight of dry SF fibers. Dry SF fibers were dissolved in 9.3 M lithium bromide solution (20% *w*/*v*) at 60 °C for 6 h and dialyzed against deionized water (ddH_2_O). The final concentration of fibroin in the aqueous silk solution was adjusted to 2 g/L, as determined by the Bradford protein assay (Thermo Fisher Scientific, Waltham, MA, USA). The turbidity of the SF aqueous solution was measured at 660 nm using a spectrophotometer (Infinite M2000pro, TECAN, Männedorf, Switzerland). The solution was casted on a flat surface of polydimethylsiloxane (PDMS), a commonly used elastomeric mold material, and was allowed to dry and form an SF-film. The SF-film was peeled off from the PDMS layer and was water vapor annealed by subjecting them to a water vapor environment in an air-evacuated desiccator to increase the ratio of beta-sheet crystallinity and hence mechanical strength. The SF-films were sterilized with 70% ethanol and UV-irradiation, soaked in a fixed dosage of IGF-1 (SRP3069, Sigma-Aldrich, St Louis, MO, USA) for 48 h at 4 °C, and washed with phosphate-buffered saline (PBS, pH 7.4) for 12 h at 4 °C.

### 2.2. Determination of the Purity and Quantity of Silk Fibroin

Sodium dodecyl sulfate-polyacrylamide gel electrophoresis (SDS-PAGE) was performed to evaluate the changes in molecular weight. Briefly, 10 µL of the degummed protein samples along with pre-stained protein ladder (BioMan Laboratories, New Taipei, Taiwan) were subjected to SDS-PAGE analysis at a constant 150 V. Gels were subsequently stained with InstantBlue coommassie protein stain (Abcam, Waltham, MA, USA) for 12 h at room temperature, and gel images were captured with an iBright FL1000 Image system (Thermo Fisher Scientific, Waltham, MA, USA). The fibroin protein band of the HD sample was quantified and normalized to the control (AD treatment) area using ImageJ 1.8.0v software (National Institute of Health, Bethesda, MD, USA).

To perform the enzyme-linked immunosorbent assay (ELISA), each well of a microwell plate was coated with 0.1 g/L of protein preparations, quantitated by the Bradford protein assay (Thermo Fisher Scientific, Waltham, MA, USA), for 8 h at 4 °C. The plate was blocked for 1 h with 5% BSA in Tris buffer-saline (pH 7.6) containing 0.1% Tween-20 (TBST), and then incubated with the anti-SF antibody and anti-silk sericin antibody (X2-Q7JYG3 and X2-O96852, both were purchased from Abmart, Berkeley, NJ, USA) separately for 2 h at 20 ± 1 °C. After washing with TBST, a horseradish peroxidase (HRP)-conjugated secondary antibody (Santa Cruz, Dallas, TX, USA) was added to the plate and incubated for 2 h at 20 ± 1 °C. This was followed by color development with the 3,3′,5,5′-TetraMethylBenzidine (TMB) single solution (Thermo Fisher Scientific, Waltham, MA, USA), and the absorbance at 450 nm was determined with a spectrophotometer (Infinite M2000pro, TECAN, Männedorf, Switzerland).

### 2.3. Characterization of Physicolchemical Properties of SF-Films

The structural features of the SF-films were evaluated using Fourier transform infrared (FTIR) spectroscopy. Information in the range of 600–4000 cm^−1^ at a resolution of 4 cm^−1^ with 64 scans was acquired with a FTIR spectrophotometer (Nicolet iS50, Thermo Fisher Scientific, Waltham, MA, USA).

### 2.4. Determination of Biocompatibility of the SF-Films

Mouse fibroblast BALB/3T3 cells were obtained from Bioresource Collection and Research Center (BCRC, Hsinchu, Taiwan). The cells were routinely grown in standard Dulbecco’s Modified Eagle’s Medium (DMEM) supplemented with 25 mM glucose and 10% fetal bovine serum under 5% CO_2_ at 37 °C to mimic diabetic conditions. To compare the effect of SF-film prepared with different degumming methods on cell viability, a SF-film of 1 cm in diameter was placed into a well of a 24-well plate, then inoculated with approximately 5 × 10^5^ cells. After incubation for 3 days, cells were treated with the LIVE/DEAD™ Viability/Cytotoxicity Kit (Thermo Fisher Scientific, Waltham, MA, USA) and examined under a fluorescence microscope according to the manufacturer’s instructions. Quantitative analysis of BALB/3T3 fibroblast grown on the SF-films was performed using 1/10 dilution of the purchased alamarBlue reagent (Thermo Fisher Scientific, Waltham, MA, USA) in DMEM for 4 h under 5% CO_2_ at 37 °C. The 100% reduced form of alamarBlue reagent, achieved by autoclaving for 10 min, was used as the 100% of cell proliferation (positive control). The fluorescence of the culture supernatant was determined using an excitation and an emission at 570 and 600 nm, respectively. The cell proliferation rate was calculated according to the formula suggested by the dye manufacturer.

### 2.5. Conjugation of IGF-1 on SF-Films

A 1 cm circular SF-film was placed on a piece of parafilm and impregnated with 100 μL of IGF-1 of a desired dosage in PBS. After incubation at 4 °C overnight, the quantity of IGF-1 bound to SF-films was determined by calculating the residual IGF-1 amount in the original solution using an IGF-1 DuoSet ELISA kit (R&D Systems, Minneapolis, MN, USA). Our result showed that a SF-film of 1 cm in diameter could absorb more than 100 pmol of IGF-1.

### 2.6. Determination of Optimal IGF-1 Dosage for Cell Migration

The cell migration assay was performed using the CytoSelect™ Wound Healing Insert (Cell Biolabs, San Diego, CA, USA) according to the manufacturer’s instructions. Briefly, the plastic insert was added into a well of a 24-well plate, then the cells were seeded at a density of 1 × 10^6^ cells/well. After incubating for 12 h, the insert was removed carefully to create a 500 μm-width cell gap for measuring migration rates of the cells. Serial dosages of IGF-1 ranging from 0.325 to 2.6 pmol in the hyperglycemic medium was tested to determine the optimal dosage of IGF-1 in vitro. The SF-films loaded with different amounts of IGF-1 were placed directly on top of the cell monolayer with a created gap in medium containing either 25 mM (regular medium) or 50 mM (hyperglycemic medium) of glucose. Wound closure was serially imaged at different times (0, 3, 6, 8, 10, and 13 days) post-scratching using an inverted light microscope (ECLIPSE Ts2-FL, Nikon, Tokyo, Japan). Autoclaved alamarBlue reagent-containing medium was used as a positive control according to the kit manufacturer’s instruction. The fluorescence of the culture supernatant, which reflected the cell metabolic activity, was determined with excitation and emission wavelengths of 570 and 600 nm, respectively. The cell proliferation rate was calculated according to the formula provided by the dye manufacturer.

### 2.7. Determination of IGF-1 Dosage Effect on Wound Healing in Diabetic Mice

The murine diabetic wound healing assay was conducted in the animal facility of National Taiwan University following the Animal Research: Reporting of In Vivo Experiments guidelines approved by the Institutional Animal Care and Use Committee and conducted according to the NIH Guide for the Care and Use of Laboratory Animals. Six-week-old leptin-receptor-deficient BKS.Cg*-Dock7^m +/+^ Lepr^db^*/JNarI female mice (*db/db*) were purchased from the National Laboratory Animal Center (Taiwan) and acclimatized in the animal facility for one week. Mice were housed in polycarbonate shoebox cages with hardwood bedding at 21 ± 1 °C under a 12 h/12 h light/dark cycle and had free access to water and food. Before wound induction, the plasma glucose levels of the mice were confirmed to be above 300 mg/dL to ensure their hyperglycemic state. After anaesthetization with isoflurane, middorsal full-thickness wounds, 8 mm in diameter including epidermis and dermis, were created. Five microliters of IGF-1 solution with a designated dosage was applied directly to the created wound and allowed to absorb for 5 min. Similarly, the SF-film with or without IGF-1 was placed directly on the wound. In either case, the wound was subsequently covered with a 3M™ Tegaderm™ I.V. Advanced Securement Dressing. Mice were divided into the following treatment groups: (A) PBS (control group), (B) 3.25 pmol IGF-1, (C) 65 pmol IGF-1, (D) SF-film (a circular film of 10 mm in diameter), (E) SF-film 3.25 pmol IGF-1 (a circular SF-film containing 3.25 pmol of IGF-1), and (F) SF-film 65 pmol IGF-1 (a circular SF-film containing 65 pmol of IGF-1). All treatments were applied to the wound beds directly and covered with a sterile adhesive film to maintain a moist environment. Wound healing was imaged after peeling off the adhesive dressing every 2–3 days using a digital camera at the same distance (30 cm) with a calibration scale on the side.

### 2.8. Histological and Immunohistochemical Analyses

Wound tissues were harvested from the sacrificed animals at the indicated time points, fixed in formalin overnight, and then embedded in paraffin. The fixed tissues were then sliced into 5 μm sections and stained using the Masson trichrome stain kit (StatLab, Lodi, CA, USA) for histological analysis. Sections were then deparaffinized, rehydrated, and subjected to sodium citrate buffer antigen retrieval. Sections were then stained with a CD31 antibody (Genetex, Irvine, CA, USA) at 1:100 dilution using an automated immunohistochemistry system (BOND-MAX, Leica, Wetzlar, Germany) and visualized at either 10× or 40× magnification, as indicated. Five stained sections from ten random fields for each group were examined under a light microscope (IM-3, OPTIKA, Ponteranica, Italy). In the measurement of the wound edge distance and epithelial gap, the shortest distance and gap were selected and used throughout the study. The Image J software was used to determine the area size of epithelial tissue. The percentage of re-epithelialization was defined as (original wound distance (i.e., 8 mm) − epithelial gap/original wound distance) × 100%. All data were the average of five independent measurements.

### 2.9. Statistical Analyses

All data are presented as mean ± SEM for each group. In vitro and in vivo studies were calculated with a minimum of *n* = 5 for each treatment. Multiple comparisons were evaluated by one-way analysis of variance (ANOVA) followed by Dunnett’s multiple comparison post hoc test to assess statistically significant differences (* *p* < 0.05, ** *p* < 0.01, and *** *p* < 0.001) between control and experimental samples. Analyses were performed using SAS-EG 7.1 software (SAS Enterprise Guide, SAS Institute Inc., Cary, NC, USA). All error bars represent the standard error of the mean (SEM).

## 3. Results

### 3.1. Manufacturing Procedure of SF-Films

**Figure 1:**
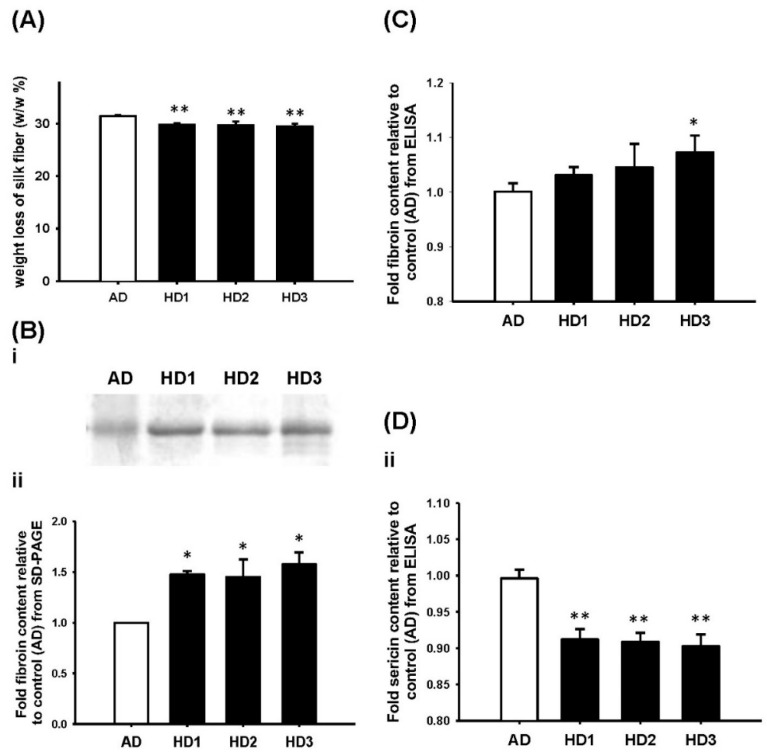
Comparison of silk fibroin purified from alkaline degumming (AD) and heat degumming for 1, 2, and 3 h (HD1, HD2, and HD3). (**A**) The weight loss of silk fibers after different treatments. (**B**) SDS-PAGE analysis of the fibroin (i) and fold content relative to control (AD) (ii) in SF prepared by HD1–3 procedures. ELISA analysis of the fold fibroin (**C**) and sericin (**D**) relative to control (AD) in SF prepared by HD1–3 procedures. Statistically significant differences between AD and HD were determined by Dunnett’s multiple comparison post hoc test. * *p* < 0.05 and ** *p* < 0.01; *n* = 4; all error bars represent the standard error of the mean.

Removal of sericin via degumming is a critical step in the production of biocompatible fibroin preparations for wound healing treatment. This study compared the properties of SF-films prepared with conventional alkaline degumming (AD) and the experimental heating degumming (HD). The average weight loss of silk during AD treatment was 31.4%, slightly higher (*p* < 0.01) than that of HD treatment (29.5–29.8%) irrespective of the treatment period (Figure 1A). The retention of fibroin light-chain protein (24–26 kDa) was 1.5–1.6-fold higher in the HD group than in the AD group, as examined using SDS-PAGE (Figure 1B). Further analysis with ELISA showed a 1.07-fold higher fibroin content in HD3 (Figure 1C) and a 0.9-fold lower sericin content in HD1–3 samples (Figure 1D) than that extracted with the AD procedure.

**Figure 2:**
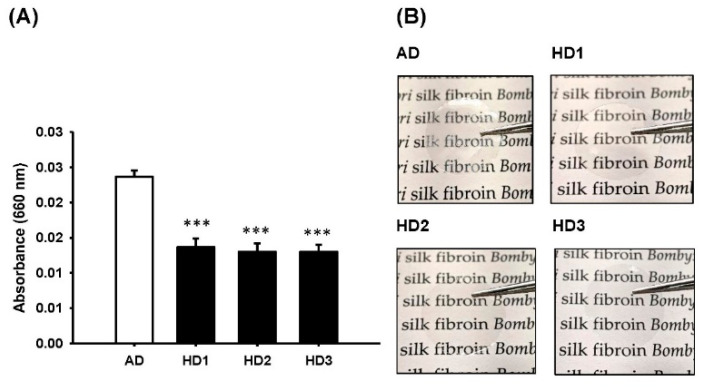
Effects of different degumming procedures on transparency of silk fibroin preparations. (**A**) Absorbance at 660 nm of the SF solution derived from alkaline degumming (AD) and heat degumming for 1, 2, and 3 h (HD1–3) procedures. (**B**) Comparison of the bright-field images of silk fibroin films. Statistically significant differences between AD and HD were determined by Dunnett’s multiple comparison post hoc test. *** *p* < 0.001; *n* = 4; all error bars represent the standard error of the mean.

**Figure 3:**
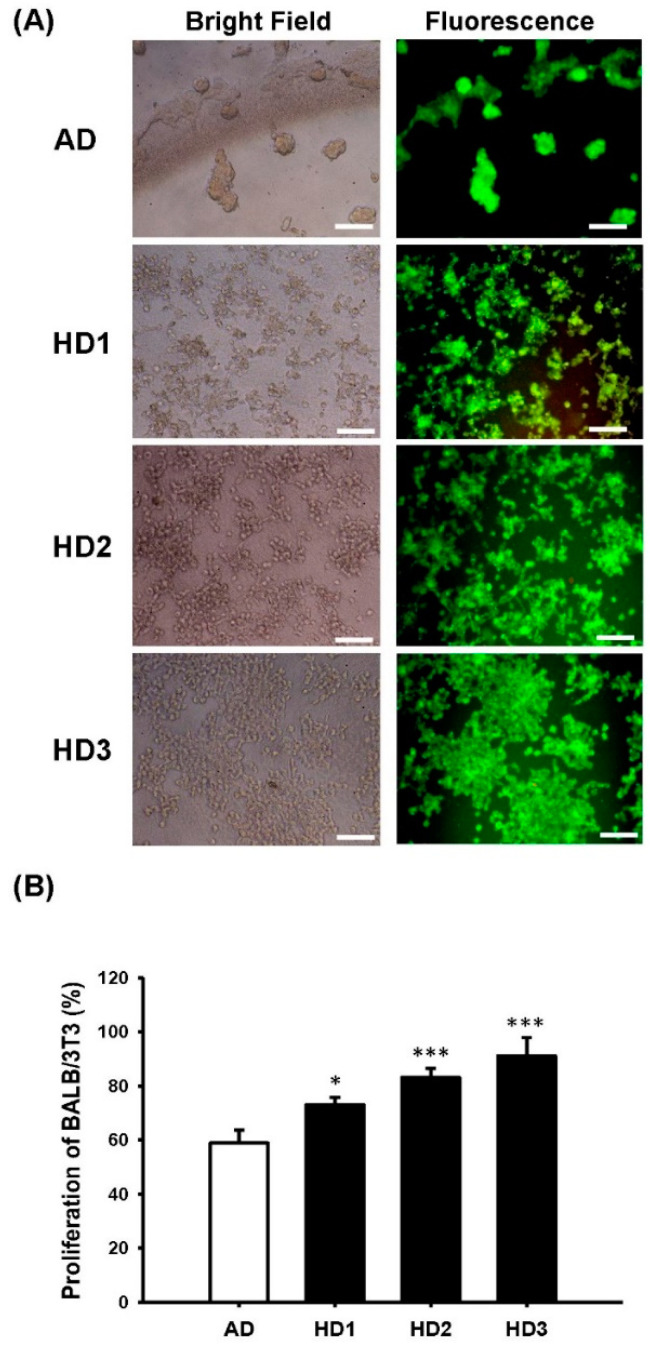
Comparison of cell viability on silk fibroin film purified from alkaline degumming (AD) and heat degumming for 1, 2, and 3 h (HD1, HD2, and HD3). (**A**) Merged images of cells grown on different SF-films for 24 h. Cells were treated with bright light and LIVE/DEAD stain under a fluorescence microscope. The polyanionic dye calcein is retained in live cells, resulting in an intense uniform green fluorescence (ex/em ~495/~515 nm). DNA intercalating dye EthD-1 enters cells, producing a bright red fluorescence in dead cells (ex/em ~495/~635 nm). Scale bars = 100 μm. (**B**) Comparison of proliferation of BALB/3T3 fibroblasts grown on the SF-films prepared from different degumming procedures. Fully reduced alamarBlue reagent of the same quantity was used as the positive control (100%). Statistically significant differences between AD and HD were determined by Dunnett’s multiple comparison post hoc test. * *p* < 0.05 and *** *p* < 0.001; *n* = 4; all error bars represent the standard error of the mean.

Film transparency, a major indicator of SF purity, is shown in Figure 2A,B. A 42% higher transparency in the HD groups than the AD group (*p* < 0.001) was noted, suggesting that SF-films derived from HD have a higher purity that may facilitate direct wound monitoring during the treatment (Figure 2B). To employ these SF-films in wound healing, we first investigated whether SF-films produced with different degumming procedures affected cell viability. As shown in Figure 3A, no cytotoxicity was detected when cells were co-incubated with any of the SF-films generated from these procedures, suggesting that the films are biocompatible. Cells on SF-film prepared using the AD method tended to aggregate into large clusters (50–100 µm), reflecting that the surface was less suitable for cell adherence. On the other hand, the cells grown on SF-films prepared using the HD method are well-dispersed (Figure 3A) and individual cells (~10 µm) could be clearly observed. Furthermore, the proliferation rate of BALB/3T3 fibroblasts grown on HD-derived SF-film was 14–32% higher than that of cells grown on AD-derived film. Among the SF-films prepared, HD3-derived film displayed the highest ability to support cell growth, indicating its suitability for wound repair uses (Figure 3B).

**Figure 4:**
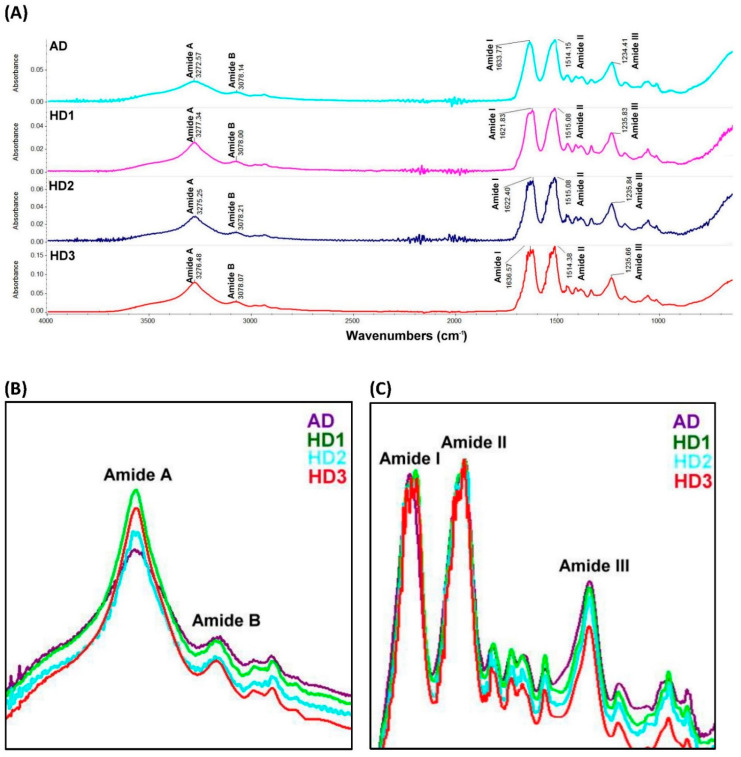
FTIR spectra of *B. mori* silk fibroin film prepared from alkaline degumming (AD) and heat degumming (HD1, HD2, and HD3) procedures. (**A**) Merged FTIR spectra of AD and HD1–3 SF-films. Overlapping spectra curves of the amide A and amide B bands (**B**), and amide I, amide II, and amide III bands (**C**) from AD and HD1–3 SF-films.

To characterize the effect of different SF preparation procedures on the protein conformation, the FTIR spectra of the generated SF-films were obtained and compared. The peak positions of amide I (C=O stretching), amide II (N-H stretching), and amide III (C-N stretching) of the SF-films, located at 1621–1636, 1514–1515, and 1234–1235 cm^−1^ respectively, were detected. In addition, we also observed the absorption band of amide A (N-H stretching) at 3272–3278 cm^−1^ and the absorption band of amide B (C-H stretching) at 3078 cm^−1^ which is a part of the double Fermi resonance with amide A among all SF-films produced with different procedures (Figure 4A). It is of interest to note that the HD-treated SF-film displayed a higher absorbance band in the amide A peak (Figure 4B) than that obtained from AD-treated SF-film. Besides, the absorbance band in amide I, II, and III was comparable between AD and HD treatments (Figure 4C). However, lower absorption bands of amide B and amide III were identified in HD3-treated SF-film than that of other treatments, which indicated lower conformations of random coil in SF-film after the HD3 procedure than other treatments. Due to its high cell proliferation and high fibroin purity, SF prepared using HD3 was used in all subsequent biological studies.

### 3.2. Effect of Different IGF-1 Dosages on BALB/3T3 Cells Grown in Hyperglycemic Medium

**Figure 5:**
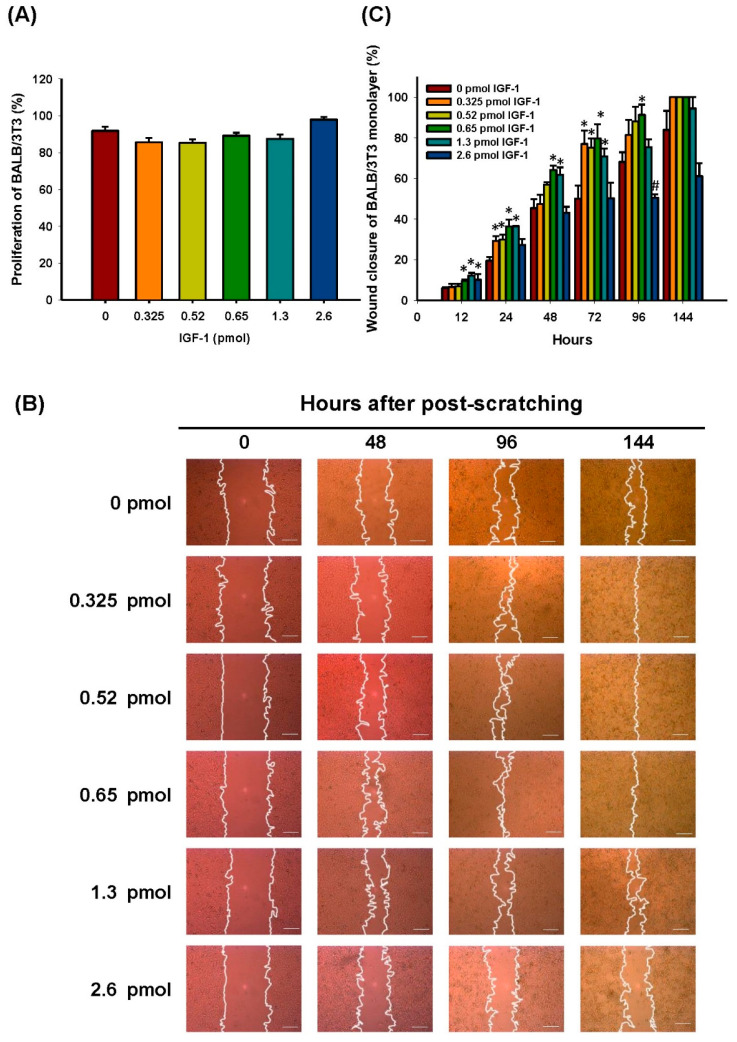
Effects of IGF-1 on BALB/3T3 fibroblast proliferation, migration, and wound healing. (**A**) Cell proliferation in the presence of different amounts of IGF-1. Fully reduced form of alamarBlue reagent of the same quantity was used as the positive control (100%). (**B**) Bright-field images of BALB/3T3 monolayer migration in the presence of different amounts of IGF-1 at different time points. Scale bars = 200 μm. (**C**) Quantitative analysis of BALB/3T3 cell monolayer wound closure improvement by IGF-1. Statistically significant differences between untreated (control) and IGF-1-treated cells were determined by Dunnett’s multiple comparison post hoc test. * *p* < 0.05 and # *p* < 0.001; *n* = 4; all error bars represent the standard error of the mean.

To identify the optimal dosage of IGF-1 for use on diabetic wounds, we investigated the effect of different dosages of IGF-1 on migration, cytotoxicity, and proliferation of BALB/3T3 fibroblasts. In the study, exogenous IGF-1 was added on top of 10% FBS to determine if higher dosages of IGF-1 could enhance cell proliferation and migration in a condition more resembling physiological conditions. The closure rates of artificially created gaps in BALB/3T3 monolayers after treatment with different IGF-1 dosages (0.325 to 2.6 pmol) for five days were determined. Initially, the effect of different IGF-1 dosages on BALB/3T3 cell growth was examined, and no significant difference was observed (Figure 5A). The addition of 0.325–1.3 pmol of IGF-1 in the hyperglycemic medium (50 mM glucose) clearly promoted cell migration after culture for 24 h. The cell monolayer gap was completely closed after 144 h in the presence of 0.325–0.65 pmol of IGF-1 (Figure 5B). The optimal dosage of IGF-1 was around 0.65 pmol, which enhanced the gap closure rate by 23% after 96 h. Higher dosages of IGF-1 inhibited cell migration. At 2.6 pmol of IGF-1, the migration rate of BALB/3T3 fibroblasts decreased significantly (17.6%, *p* < 0.001) after 96 h compared to the control (Figure 5C).

### 3.3. Effects of SF-Film Loaded with IGF-1 (SF-Film–IGF-1) on Wound Healing of BALB/3T3 Monolayers Grown in Hyperglycemic Medium

**Figure 6:**
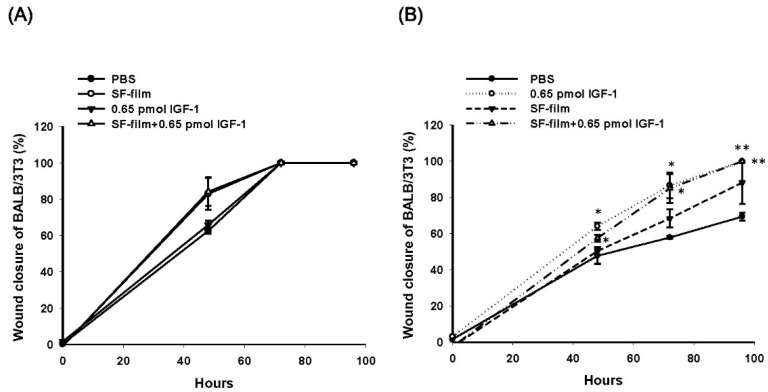
Migration of BALB/3T3 fibroblasts in response to IGF-1 (0.65 pmol), SF-film, and IGF-1-loaded SF-film (SF-film containing 0.65 pmol of IGF-1) treatments. Quantification of cell wound closure after IGF-1, SF-film, or IGF-1-loaded SF-film treatment in regular medium (**A**) or hyperglycemic medium (**B**). Statistically significant differences between the PBS and treatment groups were determined by Dunnett’s multiple comparison post hoc test. * *p* < 0.05 and ** *p* < 0.01; *n* = 4; all error bars represent the standard error of the mean.

We cultured cells in a high glucose conditions to simulate the diabetic environment. Compared to regular medium, the hyperglycemic medium inhibited wound closure of BALB/3T3 cells by 14% and 42% at 48 and 72 h, respectively (Supplementary Figure S1A). We compared the effects of 0.65 pmol of IGF-1 alone, SF-film, and SF-film containing 0.65 pmol of IGF-1 (SF-film 0.65 pmol IGF-1) on wound closure and proliferation of BALB/3T3 fibroblasts in regular and hyperglycemic medium. Interestingly, the SF-film alone was sufficient to stimulate BALB/3T3 fibroblast proliferation in regular culture medium (Supplementary Figure S1B). In regular culture medium, there was no significant difference in the wound healing of BALB/3T3 cells between any of the treatments (Figure 6A). In contrast, wound closure in hyperglycemic conditions was clearly enhanced in the presence of IGF-1 and SF-film–IGF-1. Supplementation with 0.65 pmol of IGF-1 alone for 48 h could enhance the wound closure by approximately 16% in the hyperglycemic medium. More than 10% (*p* > 0.05), 27% (*p* > 0.05), and 30% (*p* > 0.01) accelerations in wound closure were observed for SF-film containing 0.65 pmol of IGF-1 after treatment for 48, 72, and 96 h, respectively (Figure 6B).

### 3.4. In Vivo Dosage Studies of IGF-1 Loaded onto SF-Films

**Figure 7:**
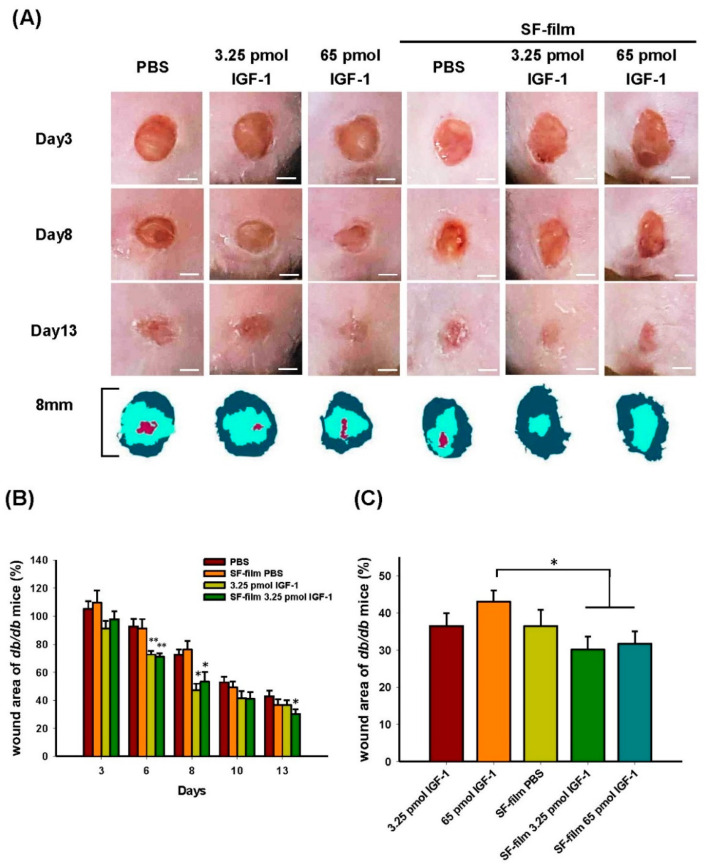
Analysis of diabetic wound healing treated with different SF-films. (**A**) Images of the wound area from day 0 to 13 post-wounding; scale bars = 5 mm. Wound closure boundaries at days 0 (dark green) and 13 (light green) and unhealed tissue at day 13 (red) are overlaid at the bottom. (**B**) Quantification of wound closure upon different treatments. Statistically significant differences between PBS and other treatments were determined by Dunnett’s multiple comparison post hoc test. * *p* < 0.05 and ** *p* < 0.01; *n* = 5; all error bars represent the standard error of the mean. (**C**) Quantification of the wound area at day 13 post-wounding after different treatments. Five stained sections for each group were evaluated and the average wound area size was estimated under a light microscope and the Image J software. Statistically significant differences between 65 pmol IGF-1 and other treatments were determined by Dunnett’s multiple comparison post hoc test. * *p* < 0.05 and ** *p* < 0.01; *n* = 5; all error bars represent the standard error of the mean.

**Figure 8:**
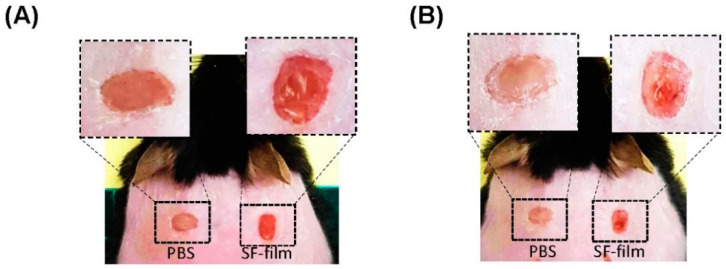
Characterization of diabetic wound tissue formation by SF-film treatment. Images of 8 mm full-thickness dorsal wounds in *db/db* mice five days (**A**) and eight days (**B**) post-wounding. The wounds were treated with PBS (left) and PBS/SF-film (right).

We investigated whether SF-film–IGF-1 promoted wound healing in diabetic mice. A mouse line deficient in leptin receptor that displayed several hallmarks of diabetes, including hyperglycemia, obesity, and impaired wound healing, was used in this study. As shown in Figure 7A, the wound closure in the experimental groups (IGF-1, SF-film, and SF-film–IGF-1) was significantly faster than that in the PBS-treated controls. The wound area of db/db mice treated with 3.25 pmol of IGF-1 either in solution or complexed with SF-film recovered by about 20% on day 6, and by 25.3% and 19.2% respectively, on day 8. The improvement in the SF-film–IGF-1 group continued and resulted in a 12.8% smaller wound area than that in the control group on day 13 (Figure 7B). The wound area of db/db mice treated with 3.25 pmol of IGF-1 was smaller than that in the control group throughout the treatment. Increasing the IGF-1 dose to 65.0 pmol, however, did not yield a better outcome (Supplementary Figure S2A). Comparable results were observed after treatment with SF-film–IGF-1 loaded with either 3.25 or 65.0 pmol of IGF-1 after eight days (Supplementary Figure S2B). Thus, it can be concluded that among all experimental groups, SF-film–IGF-1 with a relatively low dose (3.25 pmol) of IGF-1 is optimal for the treatment of diabetic wounds (Figure 7C). Interestingly, we also observed that the callus formation and micro-vessel accumulation could be observed as early as three or five days post-treatment respectively, in the SF-film only treatment group (Figure 8), which indicates that the SF-film may provide an appropriate microenvironment in tissue regeneration for diabetic wound healing.

### 3.5. Histology of Regenerated Tissue in SF-Film-Treated Diabetic Wounds

**Figure 9:**
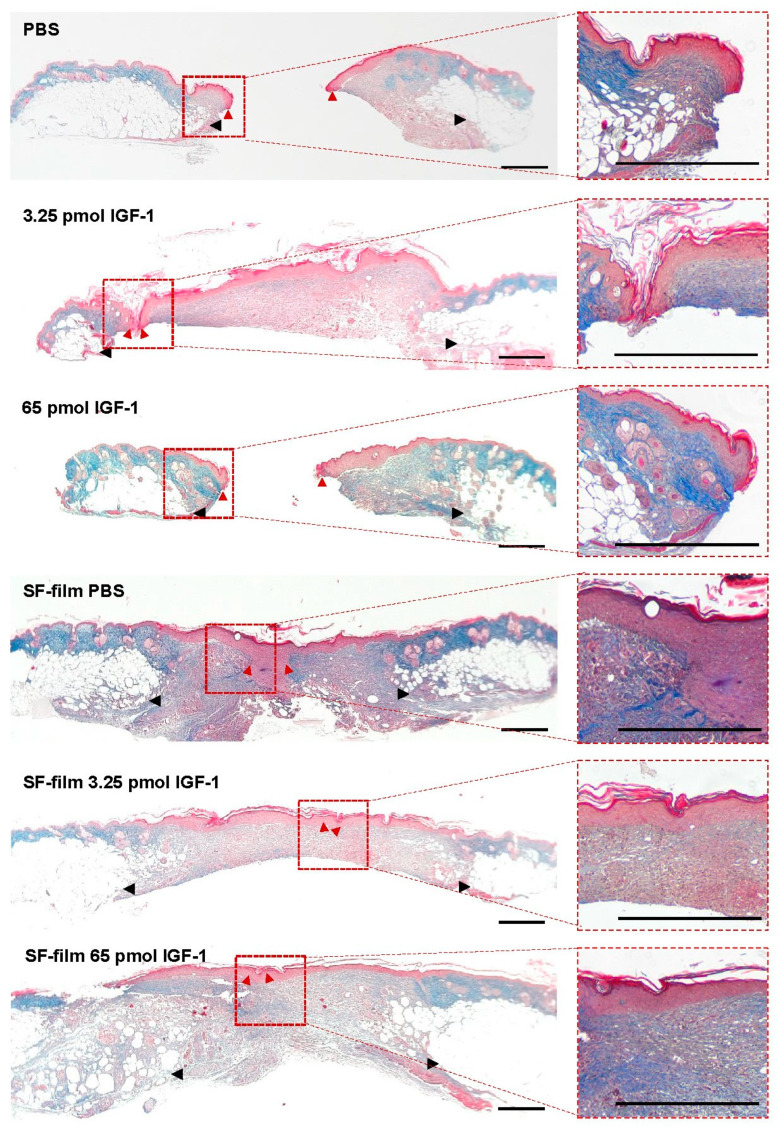
Masson’s trichrome staining of the wound area after different treatments. Tissues at day 13 post-wounding were histologically analyzed. Wound edge, black arrowheads; tips of the epithelial tongue, red arrowheads. The left panel was imaged at 10× magnification and the right panel was at 40× magnification. Scale bars = 500 μm.

**Figure 10:**
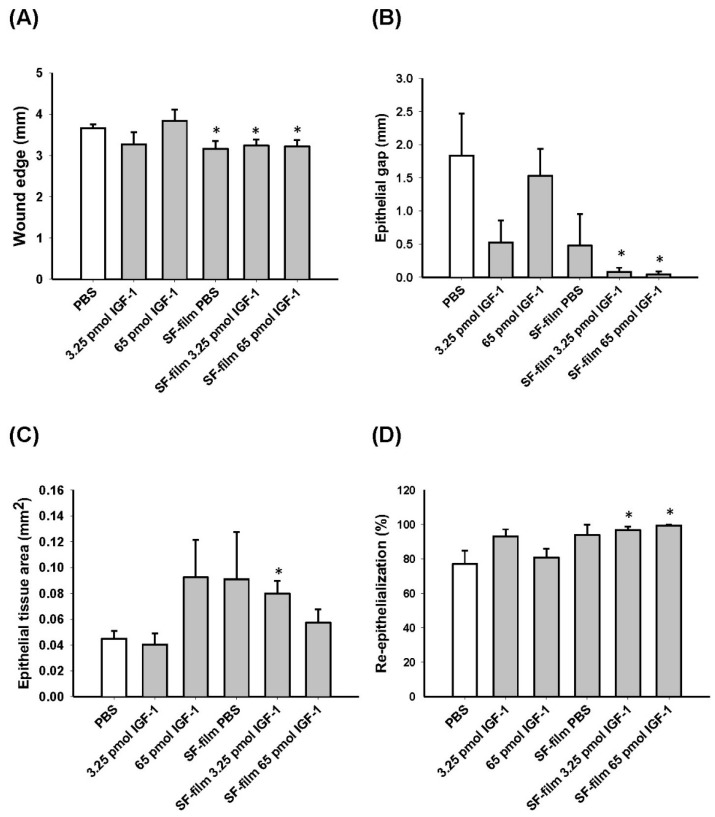
Effects of IGF-1, SF-film, and SF-film–IGF-1 on tissue regeneration in diabetic wounds. Tissues at day 13 post-wounding were histologically analyzed. Quantification of wound-edge distance (**A**), epithelial gap (**B**), epithelial tissue area (**C**), and re-epithelialization (**D**) after wound healing at day 13 post-wounding is shown. Five stained sections from 10 random fields for each group were evaluated and the average diameter was estimated under a light microscope. Significant differences between PBS and each of the treatments were determined statistically by Dunnett’s multiple comparison post hoc test. * *p* < 0.05; *n* = 5; all error bars represent the standard error of the mean.

**Figure 11:**
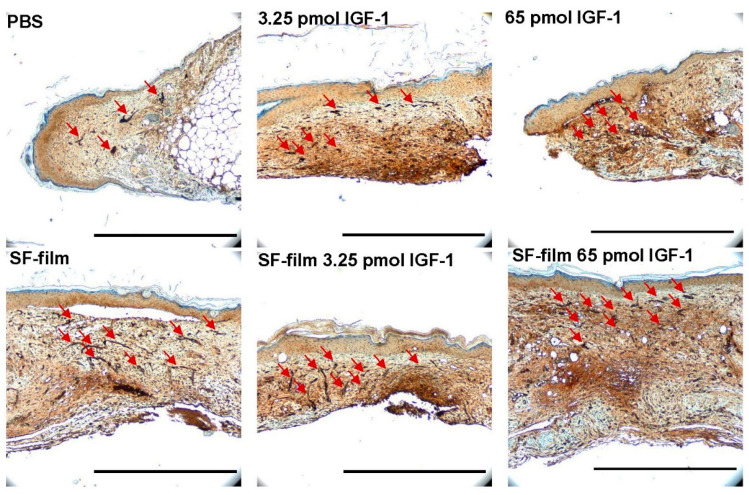
Effects of IGF-1, SF-film, and SF-film–IGF-1 on blood vessel growth in diabetic wounds. Blood vessel density in diabetic wounds after different treatments were determined immunohistologically by staining the tissue sections with an anti-human CD31 antibody. The presence of blood vessels is indicated by red arrows. The images were taken at 40× magnification. Scale bars = 500 μm.

We examined the wound histology by Masson’s trichrome staining (Figure 9). The epithelial tongue of the wound (red arrowheads) showed obviously incomplete closure in PBS alone and 65 pmol IGF-1 treatments. Although the wound was closed in 3.25 pmol IGF-1 treatments, the surface of the wound was relatively non-flat. Wound-edge distances (black arrowheads), defined as the longest distance between the two boundaries of intact skin, of SF-film- and SF-film–IGF-1-treated groups were significantly shorter than those in the other groups, demonstrating the wound healing ability of SF-films (Figure 10A). We further analyzed the epithelial gap and re-epithelialization of the regenerated tissue to assess the status of wound closure. Silk fibroin film (SF-film) loaded with either low (3.25 pmol) or high (65 pmol) doses of IGF-1 resulted in a smaller epithelial gap and better re-epithelialization (Figure 10B,D). In particular, the epithelial tissue area of the wound treated with SF-film loaded with 3.25 pmol of IGF-1 increased significantly (*p* < 0.05) compared with that of the control group (Figure 10C). On the other hand, examination of the wound histology using Masson’s trichrome staining did not reveal a significant difference between any of the SF-film-treated groups in area sizes of regenerated adipose and granulation tissues (Supplementary Figure S3A,B). Since the presence of blood vessels is an important measure of dermal restoration, we also investigated the expression of CD31, an endothelial cell-specific marker, in different experimentally treated wounds. Figure 11 shows that SF-film treatment appeared to enhance angiogenesis, as evidenced by remarkable accumulation of a large number of micro-vessels in the regenerated tissue. These results indicate that SF-film loaded with a low dosage (3.25 pmol) of IGF-1 is a useful dressing for diabetic wound treatment.

## 4. Discussion

### 4.1. Manufacturing Procedure of Silk Fibroin (SF)-Film

Raw silk fibers comprise primarily two proteins, fibroin and sericin. Sericin constitutes approximately 25–30% (*w*/*w*) of silkworm cocoons [21]. Due to its high immunogenicity, sericin must be removed before the silk material can be used in the human body. The degumming reagents, treatment time, and physical conditions, such as temperature and pressure, are key factors that affect sericin removal [37,38]. In this study, we have established a novel process involving modifications of physical degumming, dialysis, and purification procedures to improve SF purity and hence the biocompatibility of the SF-film. This HD method resulted in 1.6–1.9% higher weight loss of silk fibers, an indicator of sericin removal, than that using the conventional AD method, and as a result, 42.2–45.2% higher film transparency could be achieved. The finding is in good agreement with several earlier studies [39,40] that showed that the optical transparency of SF-films increased with a reduction of residual sericin by different degumming procedures. Significant sericin removal was also confirmed by the statistically higher amount of fibroin light-chain protein (*p* > 0.05) and lower content of sericin (*p* > 0.01) in the HD treatment group than the AD group, as determined by ELISA. Furthermore, we observed better cell growth on the fibroin film produced via the HD method than on the SF-film produced by the AD method. Our finding is consistent with previous studies which showed that a higher yield and purity of fibroin could be obtained by heating under pressure compared to sodium carbonate treatment [41,42].

This study employed FTIR to characterize the chemical bonds/functional groups on SF-film that were relevant to the physicochemical properties and drug-loading capabilities of the film [43]. This study did not observe significant wavenumber shifts in the SF-films produced by different manufacturing procedures [44,45], suggesting that no novel covalent interaction occurred. The positions of characteristic absorbance peaks for the molecular conformations of SF-film were 3280, 1630, and 1515–1530 cm^−1^ for β-sheets and 1230 cm^−1^ for random coils [46,47,48,49]. The crystallinity of secondary structure in SF derived from HD was also evaluated by calculating the absorbance peak area in FTIR spectra [50,51]. Although several reports demonstrated that different degumming procedures affected primarily the ability of SF to support cell growth, little information on molecular conformation changes was discussed [52,53]. The HD adopted in this study yielded a SF-film with a higher beta-sheet content than that of the AD procedure. Silk fibroin (SF) with a higher β-sheet content has been shown to exhibit a higher stability and longer and sustained drug release when being used in drug delivery [50,54]. Thus, these results provide strong evidence that the novel HD process could prolong the release of IGF-1 from the resulting SF-films. Another advantage of HD over AD in SF manufacturing is that HD takes less time to perform and consumes approximately 25% less reagents. Together, these findings strongly suggest that our new SF-film production method based on HD is superior to traditional methods.

### 4.2. Effects of Different Dosage and Release forms of IGF-1

Insulin-like growth factor-1 (IGF-1) is an important hormone produced by the liver that regulates secretion and physiological activities of growth hormones [55,56,57]. One major physiological function associated with IGF-1 is the stimulation of the proliferation and migration of vascular smooth muscle cells and keratinocytes [58,59,60,61]. It has been demonstrated previously that wound healing and angiogenesis could be induced by adenovirus-mediated IGF-1 over-expression ex vivo and in vivo [62]. We also previously found that IGF-1R phosphorylation in the wound bed was enhanced by IGF-1 treatment and was likely to be responsible for wound cell migration, proliferation, and wound healing [36]. Although the benefit of IGF-1 for wound healing was clearly demonstrated, there was no clinically established range of IGF-1 for wound therapy. The identification of the optimal and saturated IGF-1 dosage (3.25 and 65 pmol, respectively) in this study established a basis for future clinical application of this growth factor in wound healing.

The in vitro wound closure was statistically enhanced by more than 30% (*p* < 0.01) in the presence of IGF-1 and SF-film–IGF-1, compared with the SF-film alone or control for 96 h in hyperglycemic conditions. The results are in good agreement with a previous report [63], which demonstrated that IGF-1, rather than SF-film, played a major role in facilitating cell migration during wound healing. The fact that there was no significant difference in wound healing rate between IGF-1 and SF-film–IGF-1 in vitro also supports that the stimulatory effects of cell migration observed can be attributed primarily to IGF-1 released from the SF-films.

### 4.3. Effects of Different IGF-1 Dosage on Wound Healing

Although treatment with IGF-1 alone also produced a good wound area reduction after six days, only SF-film–IGF-1 significantly decreased the wound size at later stages of repair. Additional wound healing parameters, including epithelial gap distance, re-epithelialization degree, and epithelial tissue area, were significantly improved in the SF-film–IGF-1 group compared with the IGF-1 group. It has been demonstrated previously that IGF-1 alone only promoted the growth of myofibroblasts, but not tissue vascularization [64]. On the other hand, SF-films showed effects on acceleration of callus and micro-vessel formation, as well as a reduction of both inflammation and wound edges in diabetic wounds, irrespective of IGF-1 presence. In addition, the IGF-1 activity could be preserved for more than 30 days when conjugated with the SF-film [36]. Thus, it would not be surprising to find that SF-film–IGF-1 showed a more potent effect than free IGF-1 treatment regardless of the amount of loaded IGF-1, although the detailed mechanism remains unclear and requires further investigation. Although the results obtained in this study are very encouraging, it should be mentioned here that the murine diabetic model may not fully represent the chronic diabetic wounds in humans. In addition, the animal model only allowed short-term observation. Information concerning long-term effects and safety, which are critical in wound treatment in humans, was therefore not obtained.

Similar to the results found in cultured cells, wound repair in diabetic mice was accelerated at a low dosage of IGF-1 (3.25 pmol), showing a 49% enhancement compared to that with 65 pmol of IGF-1. Although a similar dosage-dependent effect regarding IGF-1 for tissue repair of cartilage and subchondral bone has been reported [65,66], it is not clear why a higher dosage of IGF-1 inhibits diabetic wound healing. The finding is also consistent with previous studies, which demonstrated that systemic administration of high IGF-1 dosages (0.95–1.76 μmol kg^−1^ day^−1^) to continuously stimulate wound healing was unsatisfactory [67,68], whereas IGF-I delivered locally was sufficient to improve wound healing in a diabetic model [69].

## 5. Conclusions

This study demonstrated that optimization of the SF-film manufacturing procedure and IGF-1 dosage are essential to prepare a dressing for diabetic wound healing. As far as we are aware, this study is the first report to improve the biocompatibility and crystallinity of SF-film through modification of degumming processes. We also determined that the optimal IGF-1 dosage loaded onto SF-films is 3.25 pmol, whereas a higher dose (65 pmol) showed inhibitory effects. Our results also demonstrated that SF-film plays synergistic roles with IGF-1, presumably serving as a delivery carrier for the growth factor. To sum up, SF-films produced using the modified degumming method and complexed with a low dosage (3.25 pmol) of IGF-1 is a promising dressing for wound therapy in diabetic patients.

## 6. Patents

Lin, M.-J. and Lu, M.-C. are coinventors of a patent (Taiwan patent pending number: 109138288) application pertaining to the SF-film system disclosed in the manuscript.

## Data Availability

The datasets corresponding to the current study are available from the corresponding author upon request.

## Supplementary Materials

The following are available online at https://www.mdpi.com/article/10.3390/pharmaceutics13091459/s1, Figure S1: Migration and proliferation of BALB/3T3 fibroblasts in response to insulin-like growth factor 1 (IGF-1), silk fibroin (SF)-film, and IGF-1-loaded SF-film treatment, Figure S2: Time-course analysis of diabetic wound healing after insulin-like growth factor 1 (IGF-1), silk fibroin (SF)-film, and IGF-1-loaded SF-film treatment, Figure S3: Analysis of tissue regeneration in diabetic wounds after insulin-like growth factor 1 (IGF-1)-loaded silk fibroin (SF)-film treatment.
