# Supplementary material for: An Insulin-like Growth Factor-1 Conjugated Bombyx mori Silk Fibroin Film for Diabetic Wound Healing: Fabrication, Physicochemical Property Characterization, and Dosage Optimization In Vitro and In Vivo
Source: Pharmaceutics. 2021 Sep 13;13(9):1459. doi: 10.3390/pharmaceutics13091459 (PMC8468198; doi:10.3390/pharmaceutics13091459)
Supplement: Supplementary file 1 [file pharmaceutics-13-01459-s001.zip › pharmaceutics-1328706-supplementary.pdf]

pharmaceutics

Supplementary Materials: An Insulin-Like Growth Factor-1 Conjugated Bombyx mori Silk Fibroin Film for Diabetic Wound Healing: Fabrication, Physicochemical Property Characterization, and Dosage Optimization In Vitro and In Vivo

Meng-Jin Lin, Mei-Chun Lu, Yun-Chen Chan, Yu-Fen Huang and Hwan-You Chang

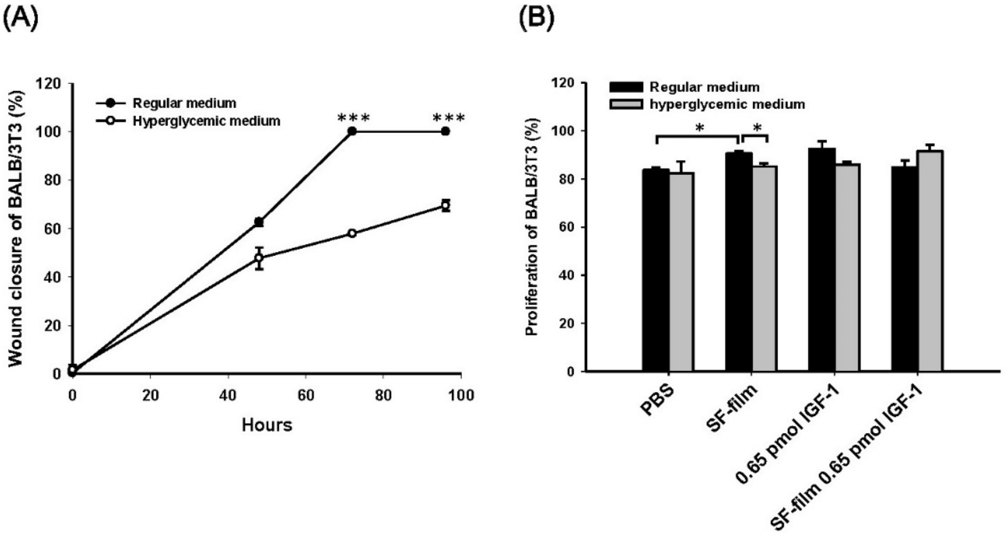

Figure S1. Migration and proliferation of BALB/3T3 fibroblasts in response to insulin-like growth factor 1 (IGF-1), silk fibroin (SF)-film, and IGF-1-loaded SF-film treatment. (**A**) Quantification of cell wound closure in regular and hyperglycemic media. Statistically significant differences between regular and hyperglycemic culture medium were determined by Dunnett's multiple comparison post hoc test. * $p < 0.05$ and *** $p < 0.001$; $n = 4$; mean ± SEM. (**B**) Cell proliferation after IGF-1 (3.25 pmol), SF-film, or IGF-1 (3.25 pmol) loaded SF-film treatment in regular or hyperglycemic medium. The 100% reduced form of alamarBlue reagent as the 100% of cell proliferation (positive control). Statistically significant differences between PBS and the other treatments were determined by Dunnett's multiple comparison post hoc test. * $p < 0.05$; $n = 4$; mean ± SEM.

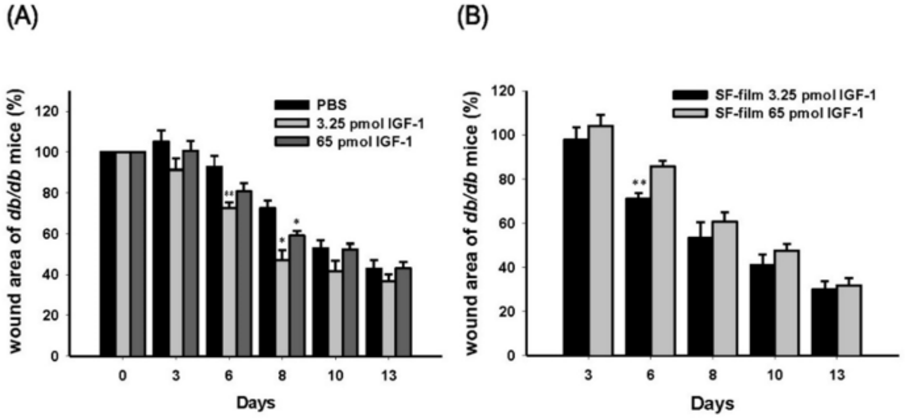

Figure S2. Time-course analysis of diabetic wound healing after insulin-like growth factor 1 (IGF-1), silk fibroin (SF)-film, and IGF-1-loaded SF-film treatment. (**A**) Quantification of wound closure from day 0 to day 13 post-wounding upon treatment with 65 pmol and 3.25 pmol IGF-1. Statistically significant differences between PBS and other IGF-1 treatments were determined by Dunnett's multiple comparison post hoc test. * $p < 0.05$, and ** $p < 0.01$; $n = 5$; mean ± SEM. (**B**) Quantification of wound closure from day 0 to day 13 post-wounding upon treatment with 65 pmol and 3.25 pmol doses of IGF-1-loaded SF-films. Five stained sections for each group were evaluated and the average di-ameter was estimated under a light microscope. Statistically significant differences between 65 pmol and 3.25 pmol dose treatments were determined by Dunnett's multiple comparison post hoc test. * $p < 0.05$, and ** $p < 0.01$; $n = 5$; mean ± SEM.

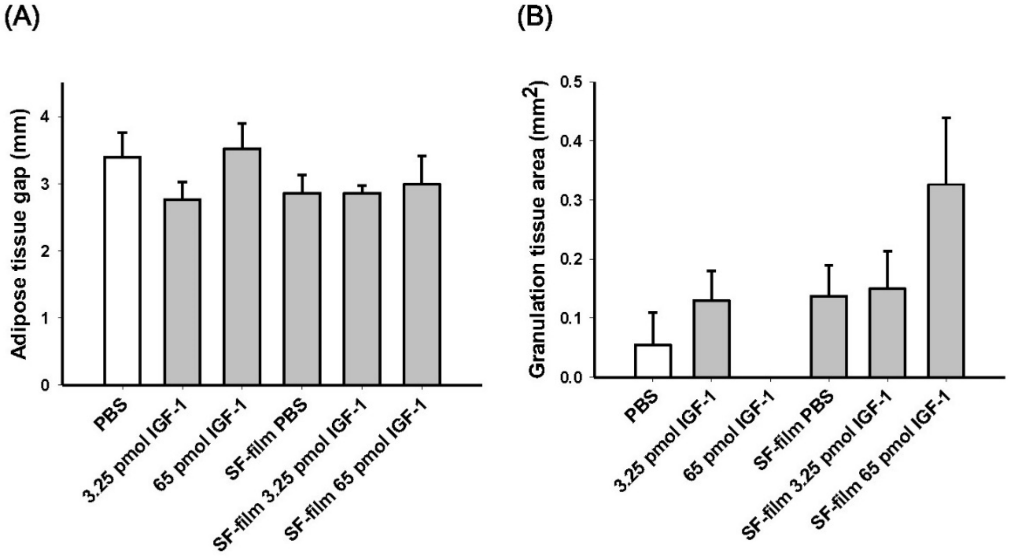

Figure S3. Analysis of tissue regeneration in diabetic wounds after insulin-like growth factor 1 (IGF-1)-loaded silk fibroin (SF)-film treatment. Tissues at day 13 post-wounding were histologically sectioned and subjected to Masson's trichrome staining. Quantification of adipose tissue gaps (**A**) and granulation tissue areas (**B**) are shown. Five stained sections from 10 random fields for each group were evaluated and the average diameter was estimated under a light microscope. No significant differences between PBS and other treatments were determined by Dunnett's multiple comparison post hoc test ($n = 5$); mean ± SEM.
